# The impact of policing and homelessness on violence experienced by women who sell sex in London: a modelling study

**DOI:** 10.1038/s41598-023-44663-w

**Published:** 2024-04-08

**Authors:** Josephine G. Walker, Jocelyn Elmes, Pippa Grenfell, Janet Eastham, Kathleen Hill, Rachel Stuart, Marie-Claude Boily, Lucy Platt, Peter Vickerman

**Affiliations:** 1 Population Health Sciences, Bristol Medical School, University of Bristol, Bristol, UK; 2 Faculty of Public Health and Policy, London School of Hygiene and Tropical Medicine, London, UK; 3 Unaffiliated, London, UK; 4 Faculty of Health and Life Sciences, Coventry University, Coventry, UK; 5 Brunel University, London, UK; 6 Department of Infectious Disease Epidemiology, Imperial College London, London, UK; 7 NIHR Health Protection Research Unit in Behavioural Science and Evaluation, University of Bristol, Bristol, UK

**Keywords:** Health services, Occupational health, Public health

## Abstract

Street-based sex workers experience considerable homelessness, drug use and police enforcement, making them vulnerable to violence from clients and other perpetrators. We used a deterministic compartmental model of street-based sex workers in London to estimate whether displacement by police and unstable housing/homelessness increases client violence. The model was parameterized and calibrated using data from a cohort study of sex workers, to the baseline percentage homeless (64%), experiencing recent client violence (72%), or recent displacement (78%), and the odds ratios of experiencing violence if homeless (1.97, 95% confidence interval 0.88–4.43) or displaced (4.79, 1.99–12.11), or of experiencing displacement if homeless (3.60, 1.59–8.17). Ending homelessness and police displacement reduces violence by 67% (95% credible interval 53–81%). The effects are non-linear; halving the rate of policing or becoming homeless reduces violence by 5.7% (3.5–10.3%) or 6.7% (3.7–10.2%), respectively. Modelled interventions have small impact with violence reducing by: 5.1% (2.1–11.4%) if the rate of becoming housed increases from 1.4 to 3.2 per person-year (Housing First initiative); 3.9% (2.4–6.9%) if the rate of policing reduces by 39% (level if recent increases had not occurred); and 10.2% (5.9–19.6%) in combination. Violence reduces by 26.5% (22.6–28.2%) if half of housed sex workers transition to indoor sex work. If homelessness decreased and policing increased as occurred during the COVID-19 pandemic in 2020, the impact on violence is negligible, decreasing by 0.7% (8.7% decrease-4.1% increase). Increasing housing and reducing policing among street-based sex workers could substantially reduce violence, but large changes are needed.

## Introduction

Some sex workers experience disproportionate sexual, physical, and emotional violence compared to non-sex workers^1^, with 19–44% ever experiencing physical violence at work and ≤ 61% experiencing intimate partner violence in the past year^2^. Risk of violence varies, influenced by structural factors such as access to housing, health, social services, poverty and work environment^2,3^. Street-based sex workers experience considerable social exclusion linked to homelessness, drug use and police enforcement that increases vulnerability to violence^4–6^. In contrast, problematic drug use, homelessness and police harassment are less common among those working indoors. As a result, sex workers working indoors usually have more control over working environments, including selection of clients, than those working on the street^3,5,7^.

In all settings, risk of violence among sex workers is increased through criminalisation. A meta-analysis found that repressive policing increased the odds of violence from clients three-fold^8^. Enforcement practices such as fines, displacement from work areas, or police raids on brothels, can reduce income, necessitating longer working hours and compromises in prices, client selection and safety^8,9^. While selling sex is legal in the UK, practices including public soliciting and working together indoors are illegal, with police also enforcing against clients, as in France and Canada^10^. Sex workers also face enforcement under laws relating to drugs, immigration and public order. Some sex worker service commissioning has shifted to prioritise assisting people to leave sex work, which can make access to services conditional^11^. These changes have been criticised by sex workers, academics and other groups as marginalising sex workers further^11^.

The East London Project (ELP), a mixed-methods participatory study, sought to measure the extent to which police enforcement practices affect sex workers’ experience of violence. The study documented how police, local authority and immigration enforcement pushed sex workers out of ‘community’ spaces where they work and live, rendering them more vulnerable to client violence and restricting access to services^12^. This and review evidence showed how enforcement practices exacerbate existing financial pressures, hindering payment of rent and reducing access to social housing or employment opportunities through having a criminal record^8,13^. Repressive policing not only created conditions in which violence was normalised, but police were also key perpetrators of recent violence against street-based sex workers (42%) alongside intimate partners (56%) and others (e.g. dealers and local residents) (67%)^3^.

Previous mathematical modelling has estimated the contribution of decriminalisation and police harassment to sex workers’ health. These models showed that decriminalisation (i.e. cessation of arrest), with reduced client violence and police harassment, could avert 33–46% of HIV cases over 10-years, while ceasing police harassment could reduce sexual and physical violence by 18% and 27%, respectively^14,15^. Neither of these models consider potential effects of a reduction in enforcement on other factors known to affect HIV or violence, including if sex workers remained discriminated against in relation to housing, drug treatment or other aspects of exclusion. We developed a mathematical model to estimate the reduction in violence that may occur if levels of policing and homelessness were reduced among sex workers, to inform supportive safety, health and welfare policies.

## Methods

We developed a mathematical model representing a population of women (primarily cis women) who sell sex that incorporates relationships between two risk factors and violence, building on data from the ELP. We simulated interventions that reduce homelessness and police displacement to estimate the resulting impact on violence. We extended the model to explore the potential impact of housed sex workers transitioning to working indoors. We focussed on client violence because the causal pathways between enforcement and client violence are more well understood than between enforcement and violence from intimate partners and others.

### Study context

We utilised data from a cohort of 90 street-based sex workers (report meeting clients outside in past 6 months) in East London (boroughs of Hackney, Newham, and Tower Hamlets) recruited by co-researchers with experience of sex work or working with sex workers, between May 2018 and September 2019^3^. Briefly, participants were recruited to an open prospective cohort study using time-location sampling of street settings and targeted sampling of online profiles, with questionnaires available in multiple languages as well as native speaker interpreters when needed. Participants were eligible if they’d provided in-person sexual services in the last three months, and were 18 years or older, of any gender, but only women were included for this modelling analysis. Three attempts were made to reach the baseline cohort for follow up, by phone, email, or street outreach; in addition, during the follow up period additional participants were recruited to increase the baseline sample size, and these were not eligible for follow up^3^. Sixty-four participants were eligible for follow up and the remaining 26 were recruited too late in the study to be followed up 6 months later. Of the 64 eligible, 35 were followed-up six months later, of whom 4 were now working indoors. No major differences were seen between the baseline and follow-up sample^3^. At baseline, participants reported high levels of violence in the past 6 months (73% reported violence from clients, 56% from intimate partners, 67% from other perpetrators, and 42% from police), current homelessness (65%), daily crack or heroin use (73%), being in arrears (57%) and depression or anxiety (71%)^3^. Most women (77%) reported being displaced from their working area by police in past 6 months.

The study was approved by the London School of Hygiene and Tropical Medicine’s Ethics Committee and the Stanmore Research Ethics Committee (IRAS ID 231206). Written informed consent was collected from all study participants prior to interview. All methods were carried out in accordance with relevant guidelines and regulations.

### Data analysis

We focus on recent (6 month) displacement by police from place of work as a key enforcement strategy that creates unsafe working conditions by forcing sex workers to move to less familiar places to work, disrupting safety strategies^8,13^. Homelessness is defined as unstable housing, including sleeping rough or living in unstable or temporary accommodation at the time of the survey^16^. Recent violence from clients is defined as either being physically attacked, held hostage, sexually assaulted, forced into degrading sexual acts, raped by clients or pressured to remove condom/or client removing condom without consent in the last 6 months. These forms of violence have been shown to be associated with police enforcement^8,17^.

We used a generalised estimating equation (GEE) model, including all baseline and follow-up data, to estimate the odds ratios (OR) for recent violence (dependent variable) if homeless or experienced recent police displacement (independent variables, reference not homeless or displaced, GEE 1). We also used a separate GEE model (GEE 2) to calculate the OR of displacement (dependent variable) if homeless (independent variable). These ORs were used as summary statistics for model parameterization.

### Model structure and parameterization

**Figure 1:**
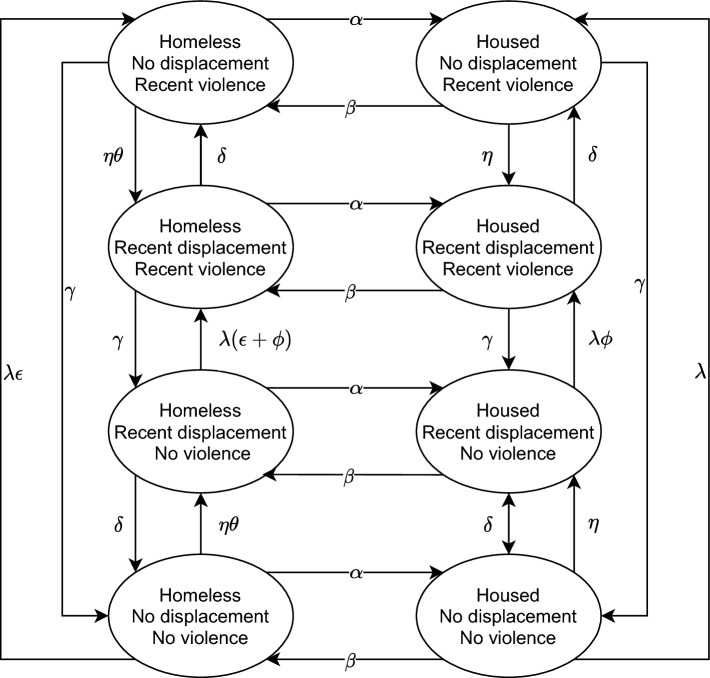
Model structure showing compartments stratified by homelessness, recent policing (displacement), and recent client violence. Arrows show transitions between compartments and Greek letters are the transition rates which are defined in Table 1.

We developed a deterministic compartmental model stratified by sex workers’ experiences of recent police displacement (in the past 6 months), recent physical or sexual violence from clients (in the past 6 months), and current homelessness (in the last 4 weeks) (Fig. 1). These categories were included in the model because they were the main modifiable predictors of client violence identified in the main data analyses and supported by the qualitative study^3,13^. We allow the rate of violence experienced by sex workers to differ by whether they have experienced recent police displacement, are currently homeless, or both. Model equations are included in the supplementary material.

The model was parameterized and calibrated using Approximate Bayesian Computation, in R (v3.6.1) using the Wegmann^18^ method (ABC-MCMC) within the package EasyABC (v1.5)^19^ (See Supplementary Methods). The model was run to equilibrium and fit to six summary statistics from the survey data, excluding four baseline observations who were missing values for displacement, client violence, or homelessness (new total of 86). This included three baseline prevalence measures among street-based sex workers: (1) percent that were currently homeless (64.0%, 95% CI 52.8–73.8%); (2) percent experiencing recent violence from clients (72.1%, 95% CI 61.2–81.0%); and (3) percent recently displaced by police (77.9%, 95% CI 67.4–85.9%). The other three summary statistics were the OR from the GEE models: the OR of violence if (4) homeless (1.97, 95% CI 0.88–4.43) or (5) displaced (4.79, 95% CI 1.99–12.11), and (6) the OR of displacement given homelessness (3.60, 95% CI 1.59–8.17). The model projections were validated by comparing the modelled proportion in each individual compartment to the observed proportion at baseline and follow-up, which were not used in fitting the model.

In order to reduce the number of uncertain parameters in the model, we made simplifying assumptions which meant that most of the variation between groups would be captured by the rate of entering, rather than leaving, each compartment. We allowed the rate of police displacement to differ by whether sex workers are homeless or not and rates of violence to differ by homelessness and whether sex workers have experienced recent policing or not. Conversely, we assumed the rates of being housed or becoming homeless were not related to experiences of recent policing or client violence. We assumed the rate of leaving recent police displacement or recent violence to be on average six months because “recent” was defined as in the past six months based on the formulated study item included in the questionnaire. These assumptions were varied in a sensitivity analysis (Supplementary Materials). We incorporated the risk of violence if homeless and/or recent police displacement as risk ratios that modified a baseline incidence of violence, with an additive effect of homelessness and police displacement because analyses did not find evidence of a multiplicative effect (not shown). Prior distribution estimations are described in the supplementary methods.

### Model outputs

#### Population attributable fraction (PAF)

To calculate the PAF of recent violence attributable to homelessness and/or displacement (proportion of violence attributable to each), we set the rate parameters for transitioning to homelessness (β) and/or displacement (η) to zero. Secondly, to explore the impact of increased police displacement among homeless sex workers on violence, we set the risk ratio of policing if homeless (θ) to one. For each, we then calculated the percentage change in the proportion of modelled individuals in the recent violence compartments at the new equilibrium. We also calculated the reduction in violence that would occur due to varying reductions in the rates of becoming homeless or recent police displacement of between 0 and 100%. We used an ANOVA model to calculate the variance explained by each of the target summary statistics (Supplementary materials).

#### Interventions

We estimated the impact of different interventions on reducing client violence against sex workers. This was implemented by changing a model parameter value and comparing the prevalence of recent violence between the intervention model and baseline model after the new model equilibrium was reached (1–2 years).

#### Reducing police displacement

We estimated the impact of reducing police displacement using publicly available policing data^20^ on stop and search encounters^21^ against women, conducted to look for controlled drugs, in the same London boroughs^12^. These data were used in the absence of better data because high levels of drug use are reported among our sample, 82/90 (91%) at baseline had used recreational drugs in the last four weeks, and 66/90 (73%) reported daily crack or heroin use^3^. Qualitative evidence and co-researcher observations suggest women are frequently stopped for drug offences^12,13^. Use of stop and search has varied over time due to government policies and priorities, with a decline from 2010/11 to 2017/2018, thereafter it increased with double the number of searches conducted in 2020/21 compared to 2017/18^22^. We conducted a breakpoint analysis (Supplementary Fig. 1) suggesting that the number of stop and search encounters would have been 39% lower at the midpoint of the cohort study period (January 2019) if the increase up to 2020 had not occurred. We therefore modelled the effect of reducing the baseline rate of policing (η) by 39%.

#### Increasing access to housing

A Housing First initiative (providing non-conditional stable housing as a starting point to end homelessness^23^) run in Leeds, UK reported that among 17 homeless street-based sex workers, 8 were provided with housing within 5 months of the programme initiating^24^. This is a rate of 1.8 transitions to housing per homeless person per year, which was added to the calibrated baseline rate of becoming housed (posterior 1.36, 95% CrI 0.40–3.25 per year) for each model run, as the intervention was supplemental to existing housing initiatives.

#### Combining both interventions

We simultaneously increased the rate of becoming housed by 1.8 per year and reduced the rate of policing by 39%.

#### Transition to indoor sex work

A secondary impact of housing may be that sex workers who are housed might transition to working indoors, which has a lower incidence of client violence compared to working outdoors (36% compared to 73% in last six months for baseline survey)^3^. This change was implemented by adapting the model to include two additional compartments to represent indoor sex workers who have or have not experienced recent violence from clients. Individuals enter these new compartments from the not homeless compartments at rate $\pi$ representing the rate of transitioning from housed, outdoor sex work to indoor sex work. Most housed sex workers in ELP work indoors (99/130, 76%), but as this does not represent the transition from street-based to indoor, we fit three scenarios such that either 5%, 50%, or 75% of housed sex workers transition to exclusively indoor sex work. We assume those moving to indoor sex work do not move back to street-based sex work. The rate of moving to the recent violence category in this group ($\kappa )$ was fit such that the proportion of indoor sex workers experiencing violence was approximately 36% as found in ELP. See the supplementary materials for details of how the parameter values were calibrated.

#### Impact of COVID-19

We evaluated the impact of two changes arising from the first national lockdown of the COVID-19 pandemic (23 March to 1 June 2020) including: (i) the provision of single-occupancy hotels rooms to nearly all street homeless and those in hostels^25^; and (ii) increases in the rate of police enforcement through anti-social behaviour orders (17,506 in Newham, Tower Hamlets and Hackney in April and May 2020 compared to 5475 in the same months in 2019)^20,26^. We modelled the impact of these temporary increases in housing accessibility and police enforcement on violence. Firstly, we set the rate of leaving homelessness (α) to 12, i.e. a person is only homeless for on average 1 month before being housed. Secondly, we increased the rate of police displacement (η) by 3.2 times. We then explored the impact these changes would have in combination.

## Results

### Model fits

**Figure 2:**
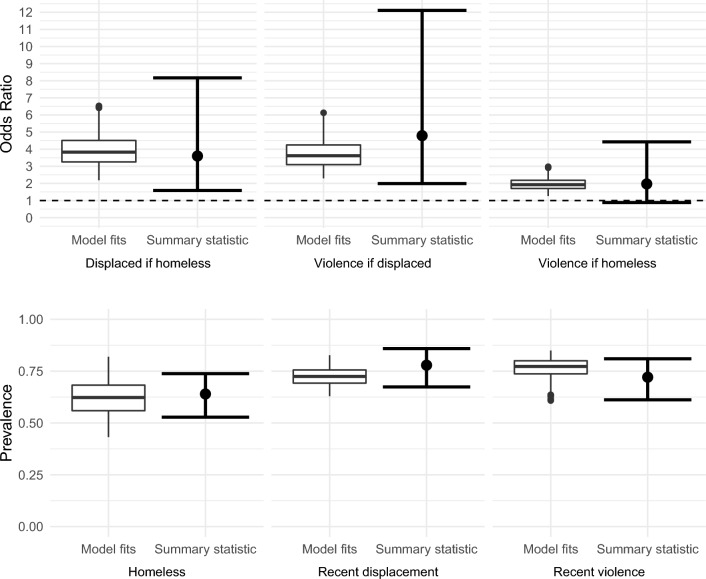
Model fits to empirical summary statistic odds ratios (top) and prevalences (bottom). Points and bars show target summary statistics and uncertainty bounds, box plots show the distribution of accepted parameter sets. Dashed line shows OR = 1.

**Figure 3:**
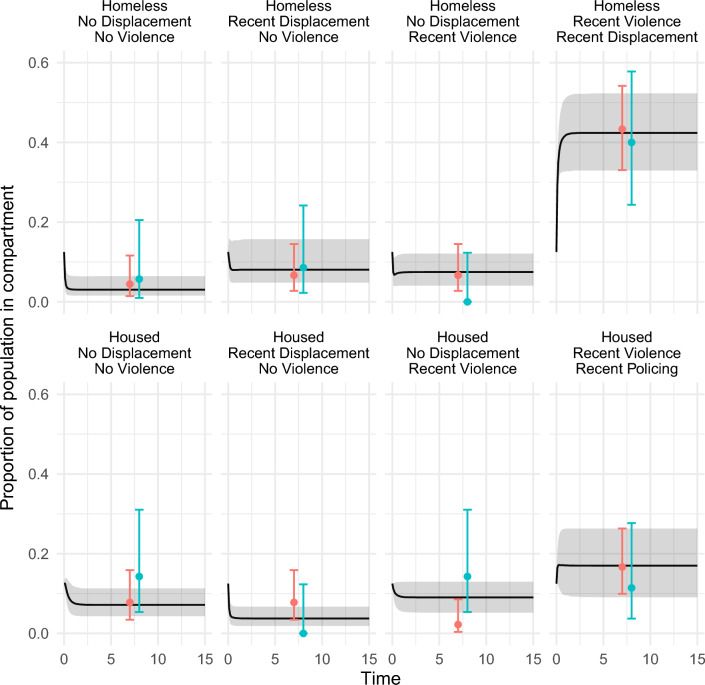
Proportion of population in each model compartment over time (black line shows median and grey shading shows 95% CrI), compared to proportion observed in the data at baseline (red points and 95% CI error bars) and follow-up (blue points and 95% CI error bars), which were not fit to. Time is in years but is just shown for illustration because the model was run until stable.

A set of 1000 parameter sets were produced through ABC-MCMC which fit the target summary statistics closely (Fig. 2), termed the baseline model fits. Modelled summary statistics from 523/1000 of the parameter sets fell within the uncertainty bounds for all six summary statistics, with all parameter sets falling within the uncertainty bounds for the OR summary statistics. The posterior distributions for the model parameters are compared to the prior distributions in Table 1 and Supplementary Fig. 2. Figure 3 shows the modelled proportion of individuals in each compartment closely fits the observed proportions, even though the model was not fit to these data.

**Table 1:** Parameters used in model and their prior distributions; rates are annual.

	Parameter	Prior distribution of annual transition rate	Prior median and 95% range	Posterior median and 95% range	Derivation from East London project data
α	Rate of leaving homelessness	– ln (1-Beta(8, 16))/0.5	0.80 (0.36–1.51)	1.36 (0.40–3.25)	7 of 23 homeless women became housed 6 months later in cohort
β	Rate of becoming homeless	– ln (1-Unif(0, 1))/0.5	1.39 (0.05–7.38)	2.26 (0.85–4.29)	Uninformative prior
γ	Rate of leaving recent violence	Fixed at 2			Violence outcome is in last 6 months so assume 6 month average duration in compartment
δ	Rate of leaving recent police displacement	Fixed at 2			Displacement outcome is in last 6 months so assume 6 month average duration in compartment
λ	Rate of violence if not homeless and not experienced recent police displacement	– ln (1-Beta(3, 3))/0.5	1.39 (0.32–3.84)	0.67 (0.30–1.21)	2 of 4 women who had not experienced recent policing or homelessness had experienced recent violence in last 6 months
ε	Risk ratio of change in violence rate if homeless	Unif (0, 10)	5.00 (0.25–9.75)	3.18 (0.32–9.32)	Estimated through calibration
ϕ	Risk ratio of change in violence rate if recent policing	Unif (0, 20)	10.0 (0.50–19.5)	15.9 (9.28–19.7)	Estimated through calibration
η	Rate of policing if not homeless	− ln (1-Beta(5, 7))/0.5	1.06 (0.37–2.36)	1.10 (0.50–2.47)	4 of 10 women who were not homeless had experienced displacement in last 6 months
θ	Risk ratio of rate of policing if homeless	Unif (0, 20)	10.0 (0.50–19.5)	11.5 (4.52–19.0)	Estimated through calibration

### Population attributable fraction

Across the baseline model fits, 77.3% (CrI 66.5–83.3%) of sex workers are in a recent violence compartment, with the largest proportion (42.4%, CrI 33.0–52.3%) in the recently displaced, homeless, and recent violence compartment (Fig. 3). When we remove homelessness, policing, or both, the proportion experiencing recent violence reduces by 29.8% (CrI 14.5–50.2%), 42.7% (CrI 23.2–77.4%), or 67.3% (CrI 53.1–81.1%), respectively (Table 2). If we remove the heightened risk of policing among the homeless the prevalence of recent violence reduces by 17.9% (CrI 8.0–38.5%).

**Table 2:** Impact of modelled interventions on proportion of population experiencing violence.

Description	Parameter change	Proportion experiencing recent violence at equilibrium	% reduction from baseline
Baseline	–	77.3% (66.5–83.3%)	–
Remove homelessness entirely	β_I_ = 0	54.0% (36.3–67.5%)	29.8% (14.5–50.2%)
Cease all police displacement	η_I_ = 0	43.7% (16.3–61.8%)	42.7% (23.2–77.4%)
Remove homelessness and cease all police displacement	β_I_ = 0; η_I_ = 0	25.2% (13.2–37.7%)	67.3% (53.1–81.1%)
Additional housing rate as seen in housing first^24^	α_I_ = α_B_ + 1.8	73.5% (59.9–80.5%)	5.1% (2.14–11.4%)
Reduce police displacement rate by 39%	η_I_ = 0.61η_B_	74.2% (63.0–80.9%)	3.9% (2.35–6.92%)
Additional housing and reduce policing together	α_I_ = α_B_ + 1.8; η_I_ = 0.61η_B_	69.6% (55.5–77.1%)	10.2% (5.91–19.6%)
Remove difference in policing between homeless and not homeless	θ_I_ = 1	63.1% (43.3–74.6%)	17.9% (8.0–38.5%)
Being housed allows 5% to transition to indoor work and reduces violence	See supplementary methods; $\pi$ and $\kappa$	75.2% (65.1–81.1%)	2.7% (2.03–3.33%)
Being housed allows 50% to transition to indoor work and reduces violence	See supplementary methods; $\pi$ and $\kappa$	56.9% (51.5–59.9%)	26.5% (22.6–28.2%)
Being housed allows 75% to transition to indoor work and reduces violence	See supplementary methods; $\pi$ and $\kappa$	46.5% (43.9–48.1%)	39.8% (34.2–42.4%)
Increase rate of housing as seen during COVID-19 lockdown	α_I_ = 12	66.8% (51.2–75.8%)	13.5% (6.91–25.2%)
Increase rate of policing as seen in COVID-19 lockdown	η_I_ = 3.2η_B_	81.9% (71.6–87.4%)	Increase by 5.7% (3.55–9.94%)
Both COVID-19 changes together	α_I_ = 12; η_I_ = 3.2η_B_	76.8% (63.6–83.8%)	0.7% decrease (8.7% decrease–4.1% increase )

Median and 95% CrI from the 1000 model fits are shown. Subscripts B and I indicate baseline and intervention parameters.

**Figure 4:**
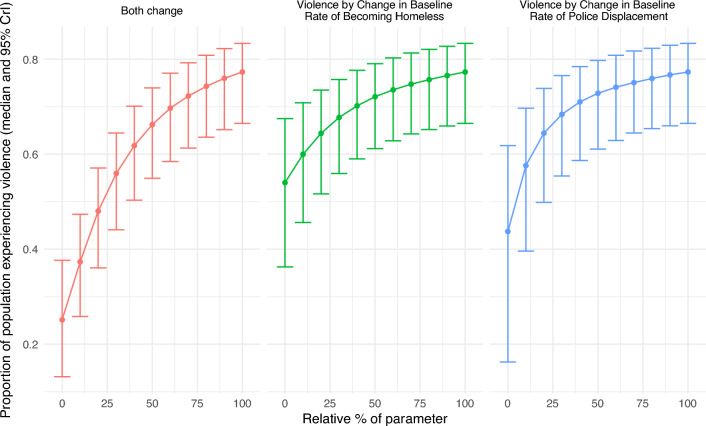
Changes in proportion of sex workers experiencing recent violence with % changes in baseline value of β (rate of transitioning to homelessness) and η (rate of experiencing police displacement) parameters, and combined. 100% denotes no change in the baseline value of each parameter.

Importantly, changes in the rate of housing and policing impact the prevalence of violence non-linearly, with the initial decline in violence due to reductions in the rate of policing or homelessness being quite small, but then the decline becomes much steeper when policing or homelessness parameters have reduced by more than 50% (Fig. 4). When displacement or homelessness parameters are reduced by half, violence reduces by 5.7% (CrI 3.5–10.3%) or 6.7% (CrI 3.7–10.2%), respectively. This non-linearity is because the baseline prevalence of violence is very high and resulting from multiple causes. In addition, due to the high prevalence of homelessness and police displacement, to reduce each by half, the rate of becoming homeless must decrease by over 70% and the rate of experiencing displacement must decrease by over 80%.

Our ANOVA analysis suggest that most of the variation in our overall PAF estimates for removing homelessness and policing together are due to uncertainty in the proportion experiencing recent violence and the OR of being displaced if homeless (Supplementary Table 1). Our sensitivity analyses found that varying the rate of leaving recent violence and homelessness had little effect on the PAF estimates (Supplementary Material).

### Interventions

Improving housing as achieved by Housing First reduces violence by 5.1% (CrI 2.1–11.4%), while it reduces by 3.9% (CrI 2.4–6.9%) if the rate of police displacement is reduced by 39%, with both combined having a synergistic effect reducing violence by 10.2% (CrI 5.9–19.6%).

If 75% of housed sex workers are able to transition to indoor sex work, then violence reduces by 39.8% (34.2–42.4%). If 50% transition to indoor sex work, violence reduces by 26.5% (CrI 22.6–28.2%), and if only 5% transition to indoor sex work, violence reduces by 2.7% (CrI 2.0–3.3%).

Lastly, providing everyone with stable housing, as occurred during the COVID-19 pandemic, reduces violence by 13.5% (CrI 6.9–25.2%). However, if the increased rate of policing seen during the COVID-19 pandemic continued, then violence would increase by 5.7% (CrI 3.6–9.9%). When these interventions are combined, the effects counteract each other, with a negligible 0.71% reduction (CrI 8.7% reduction to 4.1% increase) in violence.

## Discussion

This is the first study to simulate through mathematical modelling the effect of structural interventions in relation to housing, police displacement and transitioning to indoor sex work on violence among street-based sex workers. We found that high levels of client violence may be attributable to being displaced by the police or being homeless, with the removal of both potentially reducing violence by nearly three-quarters. However, despite their large contribution, even relatively large increases in the rate of becoming housed (more than doubling) or reductions in the rate of displacement (nearly halving) have relatively small impacts on the prevalence of violence. In the model, this is due to the very high prevalence of recent violence (77.3%), current homelessness (62.3%) and recent police displacement (72.5%) meaning that large changes are needed to have demonstrable impact on this saturated system. In addition, we do not account for other forms of marginalisation experienced by the study population, including poverty, violence from other perpetrators and incarceration, which are also likely to heighten the risk of client violence.

A key strength to our analysis is the participatory approach and its grounding in observational evidence generated through qualitative research and the cohort study of the East London Project. A simplified conceptual framework underpinning the model was developed through workshops, discussions with co-researchers and qualitative data elucidating the pathways between housing, police enforcement and violence. Limitations also exist. To ensure the model was tractable, it was necessary to focus on violence from one population group and one type of police enforcement, which does not capture the breadth of violence, the varied types of police enforcement or other aspects of exclusion experienced by street-based sex workers. Intimate partner violence, violence from strangers, and police violence are also prevalent in this population (56%, 67%, and 42%, respectively)^3^. We have not modelled the extent to which these forms of violence may be affected by different levels of police enforcement and homelessness, but they are also likely to be associated^3,13^. The modelling was also limited by the small sample in the cohort dataset and high prevalence of some risk factors, such as near universal drug use (75% reported using crack or heroin daily), which prevented any analysis of how violence might change with reductions in drug use^3^. In many cases, we only had small sample sizes from which to estimate rates of transition between different groups. This means that some priors were uninformative, increasing the importance of the calibration data points for determining these parameter values. We also assumed purely one direction of causality for the association between policing or homelessness and violence, which is an oversimplification but aligns with evidence from previous studies^2^, and was built by combining qualitative^13^ and quantitative^3^ evidence in collaboration with co-researchers with lived experience of sex work. Simplifying a complex problem into a simple causal model will not allow for a full understanding of the interactions between causal factors. Nevertheless our novel analysis showed how two key risk factors can interact to affect outcomes, and our approach can be built on as further data become available.

Furthermore, we did not incorporate heightened enforcement against sex workers based on race or sexuality, or consider other health challenges (e.g. mental health) this population face^13,27^. Most (90%) of the survey sample were UK nationals, so the model does not account for the experiences of the migrant population working in street-based sex work in East London, who are less likely to use drugs and have a very different experience of enforcement^13^.

Our modelling analyses of the potential impact of street-based sex workers becoming housed and transitioning to indoor sex work are encouraging (50% transitioning to indoor sex work leads to reduced client-violence by 26.5%) but must be interpreted with caution. While there is substantial evidence showing that indoor-based sex workers experience less violence^3,5^, in reality there are substantial barriers to transitioning from street-based sex work to indoor working^3,5^. For instance, drug use and chaotic lifestyles are less tolerated in indoor work in managed flats or saunas, or via agencies or online, while concerns about discrimination and having children taken into care present major barriers to accessing drug treatment, making it difficult for many women to manage their drug use. Such barriers need to be addressed to facilitate access to drug treatment services for those seeking treatment and support^28^. Integrated treatment programs to help street-based sex workers address their drug use as well as their mental health and trauma on their own terms are feasible in the UK^29^. Evidence also shows that transitioning to indoor sex work is feasible with low-barrier housing where sex work is tolerated and safety measures available^30^. Our model was limited by assuming that those who transition to indoor sex work do not return to the street or still sell sex on the street, which means our estimate represents a best case/optimistic scenario. Our findings support the development and expansion of such interventions that can address complex and interrelated health and social issues. However, the provision of services and support to street-based sex workers remains important, as even in settings where indoor sex work is legal, there are still sex workers who choose to, or have no option but to work on the street^31^. Sex workers also experience violence by a range of perpetrators, so interventions to reduce violence across all settings is still required^32^.

During the COVID-19 pandemic, nearly all street homeless populations and those sleeping in hostels in the UK were offered single-occupancy accommodation in hotel rooms^25^. This indicates that a large-scale intervention to address homelessness is possible. We found that providing fast access to housing for those who are homeless could reduce violence among sex workers by up to 25%. However, if this happens alongside increased policing as occurred during lockdown, the benefits are cancelled out due to the effects working in opposite directions, which demonstrates the challenges of interpreting conflicting factors that affect outcomes without modelling. Future research needs to focus on the long-term impact of the COVID-19 pandemic on sex workers’ health and precarity, not only in relation to changes in housing and enforcement but also the effects of lockdown and subsequent economic austerity and cuts to services^33,34^.

Our findings further illustrate the profound marginalisation experienced by street-based sex workers in London due to high levels of homelessness and riminalization. We find that these inequalities are synergistic and linked to poverty, drug use and incarceration, resulting in an enormous burden of violence. Our modelling analyses help us to estimate the effect of structural interventions on violence experienced by sex workers, albeit restricted to a single aspect of enforcement (displacement) and a simplified pathway from policing to violence from clients. Together with our quantitative and qualitative findings^3,13^, these analyses add to the growing body of evidence showing that decriminalisation of sex work and addressing social inequalities through provision of housing can reduce the risk of violence among sex workers^8,31^. Further studies to quantify the causal relationship between cessation of police enforcement and violence would be valuable. However the use of traditional study designs to establish causal associations would require policy changes to be implemented and raises ethical issues given the growing body of evidence showing the harms associated with all types of enforcement^3,8,35^. Evidence from countries where sex work has been decriminalised suggests discrimination and stigma remain even after decriminalisation^36^, while elsewhere police enforcement disproportionately targets sex workers who use drugs, migrants and people of colour^13,27^. Major systemic changes including tackling institutional racism and stigma are needed to substantially reduce violence experienced by this highly marginalized group.

### Supplementary Information

Supplementary Information.

## Data Availability

Data will be made available on reasonable request to Lucy Platt (data) or Josephine Walker (model code).
