# Supplementary material for: The impact of policing and homelessness on violence experienced by women who sell sex in London: a modelling study
Source: Sci Rep. 2024 Apr 8;14:8191. doi: 10.1038/s41598-023-44663-w (PMC11002010; doi:10.1038/s41598-023-44663-w)
Supplement: Supplementary file 1 — Supplementary Information. [file 41598_2023_44663_MOESM1_ESM.pdf]

Supplementary Material

The impact of policing and homelessness on violence experienced by women who sell sex in London: a modelling study

Josephine G. Walker, Jocelyn Elmes, Pippa Grenfell, Janet Eastham, Kathleen Hill, Rachel Stuart, Marie-Claude Boily, Lucy Platt, Peter Vickerman

Table of Contents

Supplementary Methods: **1**
Model equations 1
Model parameterization 2
Prior assumptions for parameters 2
Transition to indoor sex work model 3
Indoor sex work model parameterisation 3
Sensitivity analysis 4
Variance explained 4
Supplementary Results: **4**
Sensitivity analysis 4
Variance explained 5
Supplementary Table **5**
Supplementary Table 1 5
Supplementary Figures **6**
Supplementary Figure 1 6
Supplementary Figure 2 7
References **7**

Supplementary Methods:

Model equations

In the below equations, each compartment is represented as $Y_{H,V,P}(t)$ where subscript H represents homeless (1) or not homeless (0), subscript V represents recent violence (1), or no recent violence (0), and subscript P represents recent policing (1) or no recent policing (0).

$$\frac{dY_{1,1,1}}{dt} = \lambda(\varepsilon + \phi)Y_{1,0,1}(t) + \eta\theta Y_{1,1,0}(t) + \beta Y_{0,1,1}(t) - (\gamma + \delta + \alpha)Y_{1,1,1}(t) \quad \text{(Eq 1)}$$

$$\frac{dY_{1,0,1}}{dt} = \gamma Y_{1,1,1}(t) + \beta Y_{0,0,1}(t) + \eta\theta Y_{1,0,0}(t) - (\lambda(\varepsilon + \phi) + \delta + \alpha)Y_{1,0,1}(t) \quad \text{(Eq 2)}$$

$$\frac{dY_{1,0,0}}{dt} = \delta Y_{1,0,1}(t) + \gamma Y_{1,1,0}(t) + \beta Y_{0,0,0}(t) - (\lambda\varepsilon + \eta\theta + \alpha)Y_{1,0,0}(t) \quad \text{(Eq 3)}$$

$$\frac{dY_{1,1,0}}{dt} = \lambda\varepsilon Y_{1,0,0}(t) + \delta Y_{1,1,1}(t) + \beta Y_{0,1,0}(t) - (\eta\theta + \gamma + \alpha)Y_{1,1,0}(t) \quad \text{(Eq 4)}$$

$$\frac{dY_{0,1,1}}{dt} = \lambda\phi Y_{0,0,1}(t) + \eta Y_{0,1,0}(t) + \alpha Y_{1,1,1}(t) - (\delta + \gamma + \beta)Y_{0,1,1}(t) \quad \text{(Eq 5)}$$

$$\frac{dY_{0,0,1}}{dt} = \gamma Y_{0,1,1}(t) + \alpha Y_{1,0,1}(t) + \eta Y_{0,0,0}(t) - (\lambda\phi + \delta + \beta)Y_{0,0,1}(t) \quad \text{(Eq 6)}$$

$$\frac{dY_{0,0,0}}{dt} = \delta Y_{0,0,1}(t) + \gamma Y_{0,1,0}(t) + \alpha Y_{1,0,0}(t) - (\lambda + \eta + \beta)Y_{0,0,0}(t) \quad \text{(Eq 7)}$$

$$\frac{dY_{0,1,0}}{dt} = \lambda Y_{0,0,0}(t) + \delta Y_{0,1,1}(t) + \alpha Y_{1,1,0}(t) - (\eta + \gamma + \beta)Y_{0,1,0}(t) \quad \text{(Eq 8)}$$

Model parameterization

Model parameters were calibrated using Approximate Bayesian Computation with Markov Chain Monte Carlo (ABC-MCMC) such that the model outputs fit target summary statistics obtained from the survey data. Prior distributions were provided for each parameter (Table 1), and a Monte Carlo Markov chain (MCMC) was used to search this parameter space, with each step in the MCMC (set of parameter values) used to run the model and output summary statistics (S'), which were compared to a set of target summary statistics. The ABC-MCMC analysis was done in R (v3.6.1) using the Wegmann[1] method within the package EasyABC (v1.5)[2]. First, the MCMC was run for 10,000 steps, and this was used to calculate a threshold for closeness of fit between output and target summary statistics above which 1% of parameter sets would be accepted. This threshold value was then used to accept or reject parameter sets drawn in next phase of the MCMC, until 1,000 parameter sets were accepted, with every 5 steps in the chain compared to the target summary statistics and accepted or rejected. In addition, a Box-Cox transformation was applied to each of the summary statistics and partial-least squares regression was used to define components in order to reduce correlation between the summary statistics in terms of their predictive value for model parameters and use the most informative target values. For each model run, the first 15 years of simulation were discarded prior to changing parameters to ensure models had reached equilibrium before introducing changes.

Prior assumptions for parameters

Prior distributions for the proportion that transition between groups were based on an uninformative Beta(1,1) prior (equivalent to a uniform distribution between 0 and 1). The Beta distribution was updated with observed data from the cohort study by adding the number that transitioned to the first shape parameter and the number that didn’t transition to the second shape parameter. This results in a prior distribution between 0 and 1 that is centred on the observed proportion that transitioned and the standard deviation of the distribution decreases as the sample size of the observations increase. The parameters representing the proportion transitioning are then converted to annual transition rates within the model by $-\ln\,(1 - proportion)/0.5$.

This method of prior estimation was used for three parameters based on the cohort data: the rate of transitioning to recent violence category if not recently displaced or homeless (λ), the rate of transitioning to recent policing category if not homeless (η), and the rate of leaving homelessness (α). For λ and η, we looked only at individuals who were in the relevant risk category (not recently displaced nor homeless, or not homeless, respectively) at both baseline and follow. Of these, we calculated the proportion who reported the relevant outcome (recent violence or recent police displacement), at follow up. This represents the proportion who experienced the outcome while remaining consistently in the same (lower) risk category. For the rate of leaving homelessness, we calculated the number of participants who transitioned from homelessness to housing between the baseline and follow up surveys.

Transition to indoor sex work model

We adapted the baseline model to include two additional compartments representing indoor sex workers who have or have not experienced recent violence from clients.
The below equations describe the alternative model structure as adapted from Eq 1-8. The changes were implemented by adding additional compartments which represent indoor sex workers who have (Z_1) or have not (Z_0) experienced physical or sexual violence from clients in the past 6 months. These new compartments are linked to the not homeless compartments (New Eq 5b-8b) by the parameter π which represents the annual rate of transition from housed, street-based sex work, to indoor sex work, regardless of policing status. For simplicity, we assume those moving to indoor sex work do not move back to outdoor sex work. As in the main model, the average duration in Z_1 is assumed to be 6 months, so the rate of transition to Z_0 is fixed at 2. The rate of transition from Z_0 to Z_1 is defined as κ.

$$\frac{dY_{0,1,1}}{dt} = \lambda\phi Y_{0,0,1}(t) + \eta Y_{0,1,0}(t) + \alpha Y_{1,1,1}(t) - (\delta + \gamma + \beta + \pi) Y_{0,1,1}(t) \quad \text{(Eq 5b)}$$

$$\frac{dY_{0,0,1}}{dt} = \gamma Y_{0,1,1}(t) + \alpha Y_{1,0,1}(t) + \eta Y_{0,0,0}(t) - (\lambda\phi + \delta + \beta + \pi) Y_{0,0,1}(t) \quad \text{(Eq 6b)}$$

$$\frac{dY_{0,0,0}}{dt} = \delta Y_{0,0,1}(t) + \gamma Y_{0,1,0}(t) + \alpha Y_{1,0,0}(t) - (\lambda + \eta + \beta + \pi) Y_{0,0,0}(t) \quad \text{(Eq 7b)}$$

$$\frac{dY_{0,1,0}}{dt} = \lambda Y_{0,0,0}(t) + \delta Y_{0,1,1}(t) + \alpha Y_{1,1,0}(t) - (\eta + \gamma + \beta + \pi) Y_{0,1,0}(t) \quad \text{(Eq 8b)}$$

$$\frac{dZ_1}{dt} = \pi\left(Y_{0,1,1}(t) + Y_{0,1,0}(t)\right) - (2)Z_1(t) + (\kappa)Z_0(t) \quad \text{(Eq 9)}$$

$$\frac{dZ_0}{dt} = \pi\left(Y_{0,0,1}(t) + Y_{0,0,0}(t)\right) - (\kappa)Z_0(t) + (2)Z_1(t) \quad \text{(Eq 10)}$$

Indoor sex work model parameterisation

For each of the 1000 parameter sets from the baseline model, we estimated two additional parameters. First, as the possibility of transitioning to indoor sex work is uncertain, we set a target value of 5%, 50%, or 75% for the proportion of the total population who transition to indoor sex work (Z_0 + Z_1 at equilibrium). We then fixed κ at 0 and tested a sequence of values for π ranging from 0.001 to 0.1 in increments of 0.001, and selected the value of π

which minimized the distance from 5%, 50%, or 75% for the proportion of modelled individuals in Z compartments. The range was of tested parameters was expanded until the target values could be reached. By incrementing parameters rather than fitting precisely to the target values, we allow for an uncertainty range around the target proportions.

We then used a similar method to conduct a second step fitting procedure for κ. In each parameter set for the 5%, 50%, and 75% scenarios, we set a target value for the proportion of indoor sex workers experiencing recent violence $Z_1/(Z_0 + Z_1)$ to be 36%, as observed in the East London Project. The sequence of values tested for κ ranged from 0.1 to 0.6 in increments of 0.01.

The resulting parameter distributions are: for the 5% target, π ranges from 0.002-0.005 and κ is 0.42-0.43 resulting in 4.0-6.6% transitioning to indoor sex work, with 35.6-36.4% of indoor sex workers experiencing recent violence; for the 50% target, π ranges from 0.020-0.063 and κ is always 0.43 resulting in 49.2-50.8% transitioning to indoor sex work, with 36.1-36.3% of indoor sex workers experiencing recent violence; for the 75% target, π ranges from 0.040-0.129 and κ is 0.43, 74.7-75.3% transition to indoor sex work, and 36.1-36.2% of indoor sex workers experiencing recent violence.

Sensitivity analysis

An alternative version of the model was tested by changing the fixed rate of leaving recent violence (γ) and rate of leaving recent displacement (δ) to 1 or 2.5 (compared to baseline with both values fixed at 2). The model was re-fit allowing all parameters to adjust alongside the new values for the fixed parameters and the PAF was calculated as in the main analysis. We tested using larger values, however setting the parameters to 3 or greater resulted in failure of the ABC fitting process.

Variance explained

To assess what drives differences in each PAF value, we conducted linear regression analyses with each of the PAF calculations (remove homelessness, remove displacement, remove both) as dependent variables, and the six target summary statistics as independent variables, included in the model in the order they are listed in the main text methods. We then used the anova() function in R to calculate the variance explained by each predictor as the incremental sum of squares.

Supplementary Results:

Sensitivity analysis

Alternative model fits setting the rates of leaving recent violence and recent displacement to 1 resulted in only 238 out of 1000 parameter sets fitting within the uncertainty bounds of all target summary statistics. Under this scenario, the proportion reduction in violence when homelessness is eliminated increased to 40.5% (95% CrI 18.1-65.0%) compared to 29.8% (95% CrI 14.5-50.2%) in the base case, while the reduction in violence with removal of police displacement remained similar at 40.8% (95% CrI 22.9-79.6%) compared to 42.7% (95% CrI 23.2-77.4%) in the base case. For removing both homelessness and displacement, the reduction in violence again was similar, at 72.9% (95% CrI 53.9-86.3%) compared to 67.3% (95% CrI 53.1-81.1%) in the base case. Increasing the rates of leaving recent violence and

recent displacement to 2.5 resulted in 543/1000 parameter sets fitting within the uncertainty bounds of all target summary statistics. The reduction in violence when homelessness or displacement are removed both decreases slightly compared to the base case, with 25.3% (95% CrI 7.7-43.0%) reduction for homelessness and 39.0% (21.1-75.2%) for displacement. When both are removed the result is similar, with a 64.9% (48.2-77.9%) reduction in violence.

Variance explained

The proportional incremental variance explained for each of the PAF scenarios is shown in Supplementary Table 1.

Supplementary Table

Supplementary Table 1

Proportional incremental variance explained by summary statistics for each PAF scenario (ANOVA analysis)

**Summary statistic**	**Remove Homelessness**	**Remove Displacement**	**Remove Both**
Proportion Homeless	10.4%	0.924%	5.30%
Proportion recent violence	8.68%	9.82%	30.2%
Proportion recent displacement	8.52%	1.73%	10.7%
OR violence if homeless	2.89%	34.2%	10.8%
OR violence if displaced	0.916%	28.9%	0.574%
OR displaced if homeless	0.142%	0.83%	17.1%
Residuals	68.41%	23.6%	25.3%

Supplementary Figures

Supplementary Figure 1

Total monthly stop and search events due to suspected controlled drugs, for the three London boroughs of Newham, Tower Hamlets, and Hackney. The solid line shows the breakpoint analysis linear regression, dashed line shows the forward projection of the line prior to the breakpoint. Horizontal dotted lines represent the start and end dates of the East London Project cohort data collection. Dots shown in red are two outlier points which were excluded from the breakpoint regression.

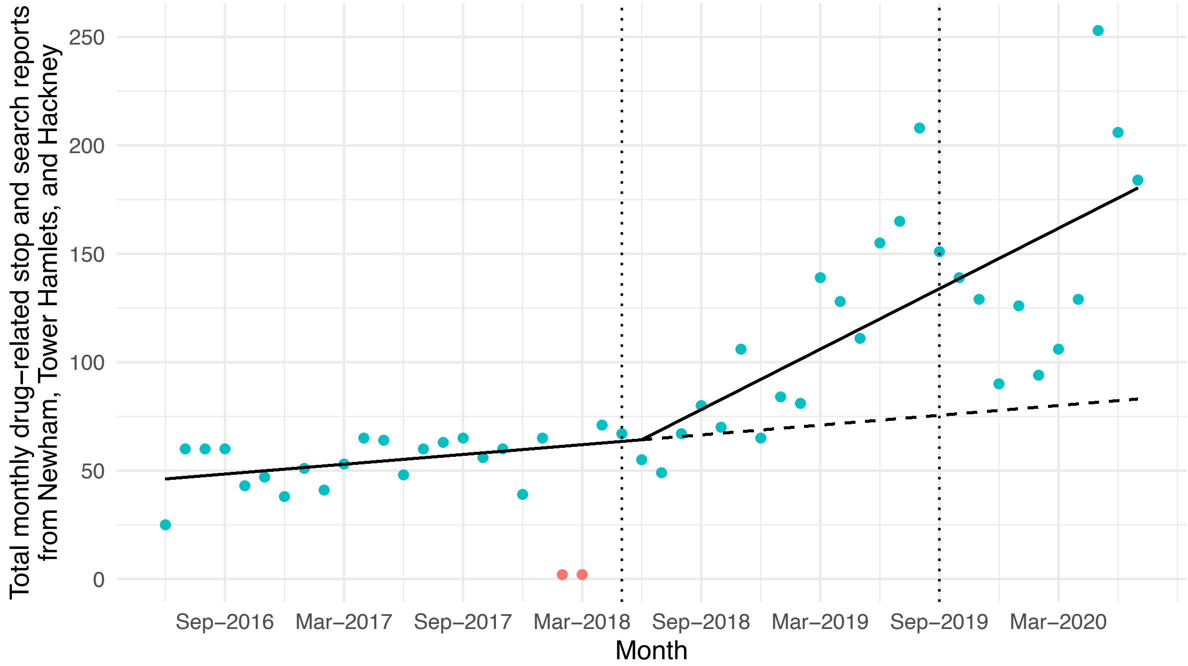

Supplementary Figure 2

Prior and posterior distributions for the seven sampled parameters showing distributions sampled before transformation to rates; Beta priors (top) and uniform priors (bottom).

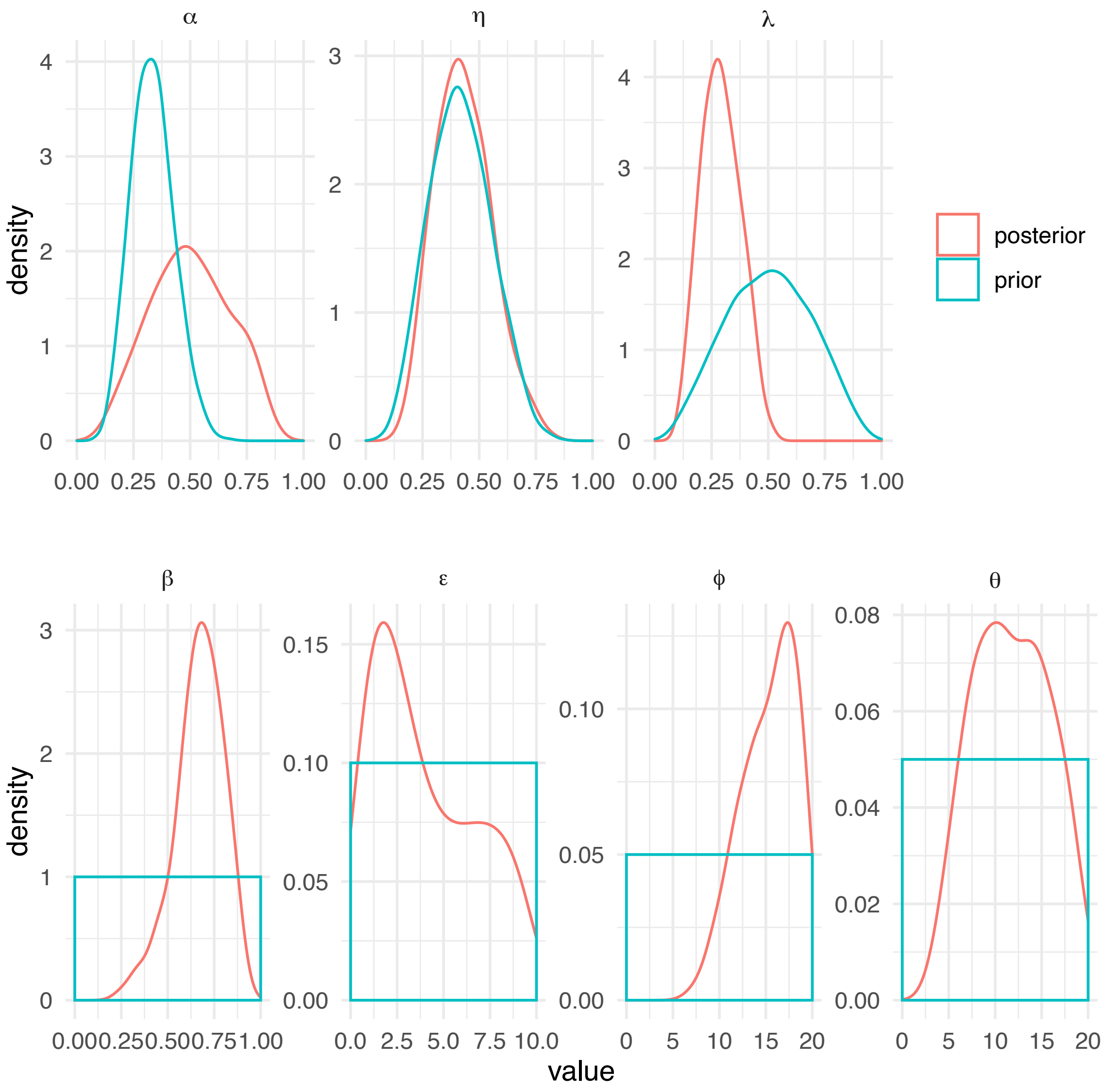

References

1. Wegmann D, Leuenberger C, Excoffier L. Efficient approximate Bayesian computation coupled with Markov chain Monte Carlo without likelihood. *Genetics* 2009; **182**(4): 1207-18.

2. Jabot F, Faure T, Dumoulin N. EasyABC: performing efficient approximate Bayesian computation sampling schemes using R. *Methods in Ecology and Evolution* 2013; **4**(7): 684-7.
